# Fermented Supernatants of *Lactobacillus plantarum* GKM3 and *Bifidobacterium lactis* GKK2 Protect against Protein Glycation and Inhibit Glycated Protein Ligation

**DOI:** 10.3390/nu15020277

**Published:** 2023-01-05

**Authors:** Shih-Wei Lin, Chi-Hao Wu, Ya-Chien Jao, You-Shan Tsai, Yen-Lien Chen, Chin-Chu Chen, Tony J. Fang, Chi-Fai Chau

**Affiliations:** 1 Department of Food Science and Biotechnology, National Chung Hsing University, Taichung 40227, Taiwan; 2 Graduate Programs of Nutrition Science, School of Life Science, National Taiwan Normal University, Taipei 106209, Taiwan; 3 Product and Process Research Center, Food Industry Research and Development Institute, Hsinchu 30062, Taiwan; 4 Biotech Research Institute, Grape King Bio Ltd., Taoyuan 32542, Taiwan; 5 Institute of Food Science and Technology, National Taiwan University, Taipei 10617, Taiwan; 6 Department of Food Science, Nutrition and Nutraceutical Biotechnology, Shih Chien University, Taipei 10462, Taiwan; 7 Department of Bioscience Technology, Chung Yuan Christian University, Taoyuan 32023, Taiwan

**Keywords:** aging, protein glycation, *Lactobacillus plantarum* GKM3, *Bifidobacterium lactis* GKK2, fermented supernatants, the receptor for advanced glycation end products (RAGE)

## Abstract

With age, protein glycation in organisms increases continuously. Evidence from many studies shows that the accumulation of glycated protein is highly correlated with biological aging and the development of aging-related diseases, so developing a dietary agent to attenuate protein glycation is very meaningful. Previous studies have indicated that lactic acid bacteria-fermented products have diverse biological activities especially in anti-aging, so this study was aimed to investigate the inhibitory effect of the fermented supernatants of *Lactobacillus plantarum* GKM3 (GKM3) and *Bifidobacterium lactis* GKK2 (GKK2) on protein glycation. The results show that GKM3- and GKK2-fermented supernatants can significantly inhibit protein glycation by capturing a glycation agent (methylglyoxal) and/or protecting functional groups in protein against methylglyoxal-induced responses. GKM3- and GKK2-fermented supernatants can also significantly inhibit the binding of glycated proteins to the receptor for advanced glycation end products (RAGE). In conclusion, lactic acid bacteria fermentation products have the potential to attenuate biological aging by inhibiting protein glycation.

## 1. Introduction

Aging is often recognized as a progressive degeneration in physiological capacity, which may be a consequence of reactive oxygen species (ROS)-/reactive carbonyl species (RCS)-mediated biological events [1,2]. In this regard, RCS-induced protein glycation and the derived accumulation of advanced glycation end products (AGEs) during the aging process have received great attention, because these events may be the key bridge connecting aging with different human diseases such as atherosclerosis, diabetes, or neurodegenerative symptoms [3]. The content of AGEs mainly comes from the glycation reaction in the body, followed by diet, and is highly correlated with the concentration of glucose in the body and the metabolic response of glucose; therefore, it is interesting to investigate health-promoting dietary intervention that can disrupt RCS-induced glycation and AGE formation to decrease the onset and progression of aging-related diseases.

The glycation reaction can be divided into three main stages. The first stage is the interaction between carbohydrate molecules and substances with amine groups to form an unstable Schiff base, which further convert into the enaminol intermediate and then rearrange to the more stable Amadori products (e.g., ε-amino-lysine, ketoamine) [4]. The reactions in this phase are all reversible, but the formation of the Amadori products from the enaminol intermediate is faster than the opposite reaction. Therefore, the Amadori products are accumulated. The second stage is the degradation of Amadori products to produce highly active carbonyl compounds such as glyoxal (GO), methylglyoxal (MGO), or 3-deoxyglucosone (3-DG). During the production of these carbonyl compounds, the metal ions or oxygen are involved as catalysts. The third stage is the process in which active molecules interact with proteins or amino acids in the middle stage of glycation to generate stable substances (so-called glycated proteins or glycated molecules). Generally, the production of AGEs is irreversible. These AGEs usually contain arginine and lysine residues; therefore, they bind to the receptors for highly glycated end products (RAGE) located on the cell membrane triggering the cell physiological mechanism such as pro-inflammatory cytokines in the pathogenesis of chronic diseases [5].

The use of food with anti-glycation activity represents a potential strategy to treat and prevent AGE-related diseases. For example, the phenolic compounds from citrus fruits have been reported with an ability to inhibit AGE formation [6]. The isoflavones from legumes also presented with anti-glycation capacity in an in vitro experiment [7]. These anti-glycation compounds from food material usually come from enzymatic or microbial fermentation in the human gut. Therefore, focusing on gut microbiota or specific microbial supplements with anti-glycation effects might be one of the solutions for disease prevention.

Lactic acid bacteria (LAB) have been widely demonstrated as probiotics, which have several health benefits such as assistance for body weight control, the homeostasis of gut microbiota, immune modulation, and the prevention of metabolic diseases and cancer [8,9]. Our research group has demonstrated that LAB, including *Lactobacillus plantarum* GKM3 (GKM3) and *Bifidobacterium lactis* GKK2 (GKK2), also have great anti-aging properties [10,11]. In the senescence-accelerated prone mice P8 (SAMP8) animal model, the administration of GKM3 (1.0 × 109 cfu/kg B.W./day) effectively decreased TBARS and 8-OHdG levels in mice brains as well as the related features of cognitive impairment from the passive and active avoidance test, suggesting that GKM3 has great potential for delaying the oxidative-stress-related aging process [11]. The administration of GKK2 was also found to delay the progression of aging in SAMP8 mice as evidenced by the score of senescence from Takeda’s grading method. Furthermore, decreased age-related muscle loss was found in GKK2-treated mice when compared to the vehicle controls [10]. Because anti-glycation characteristics such as anti-oxidative and anti-inflammatory activities were also found in other GKM3 and GKK2 studies [12,13,14], the present study developed an in vitro methylglyoxal-induced bovine serum albumin (MG-BSA) model mimicking protein glycation to clarify whether the anti-aging actions of GKM3 and GKK2 fermentate were partially inhibiting RCS-induced glycation.

## 2. Materials and Methods

### 2.1. Fermented Supernatant Samples

*Lactobacillus plantarum* GKM3 (BCRC 910787) and *Bifidobacterium lactis* GKK2 (BCRC 910826) were isolated from fresh vegetable and infant feces, respectively. Fermented supernatants of *L. plantarum* GKM3 (GKM3) and *B. lactis* GKK2 (GKK2) were provided by Grape King Bio Ltd. (Taoyuan, Taiwan). In general, bacteria strains GKM3 and GKK2 were cultured in a 15-tons fermenter, respectively, at 37 ℃ for 16 h with containing an 80% medium (5% glucose, 2.0% yeast extract, 0.05% MgSO_4_, 0.1% K_2_HPO_4_, and 0.1% Tween 80) under pH control at 6.0. During the fermentation of the strain GKK2, the addition of CO_2_ gas was needed. After incubation, the fermented supernatants were collected and centrifuged. The pH values of the fermented supernatants were then adjusted to 6.8–7.2 and these supernatants were heated at 121 °C for 1 min. Finally, GKM3- and GKK2-fermented supernatants were stored at −30 °C for study. The protein contents of the GKM3- and GKK2-fermented supernatants were 7 ng/mL and 28 ng/mL, respectively.

### 2.2. Protein Glycation Assays

Protein glycation was carried out by the reaction of a highly reactive dicarbonyl-methylglyoxal (MG; 25 mM) and bovine serum albumin (BSA, the most abundant albumin in the mammalian circulatory system; 5 mg/mL) at 37 °C for 7 days. GKM3- and GKK2-fermented supernatants (50 μL/mL) were added to the in vitro system of protein glycation as mentioned above to investigate their inhibitory effects on protein glycation. The control groups are BSA incubated with the vehicle, GKM3- or GKK2-fermented supernatants without MG addition. At the end of the first day of the reaction, an aliquot of the reaction solution was subjected to an analysis of the Amadori products that were the main products in the early protein glycation stage using a nitroblue tetrazolium (NBT) reagent as previously described [15]. In the NBT assay, the basic carbonate buffer containing NBT was mixed with the sample for 15 min. Since the glucose reduced NBT, the colorless NBT became to a deep blue, and the absorbance at 530 nm was then determined.

At the end of the reaction, 10% sodium dodecyl sulfate polyacrylamide gel electrophoresis (SDS-PAGE) and 3-D fluorescence spectroscopy were used to analyze the molecular characteristics of the glycated protein in each group according to the previous methods [16,17,18]. Specific functional groups, including free ε-amino groups measured by trinitrobenzenesulfonic acid (TNBS) [19] and carbonyl content measured by 2,4-dinitrophenylhydrazine (DNPH) [20] were further determined in these glycated proteins in order to clarify the underlying mechanism of the actions of GKM3- and GKK2-fermented supernatants in protein glycation. According to the previous study [21], an aliquot of the reaction solution was also taken for the determination of the content of MG-derived glycation products (argpyrimidine) in each group using a fluorescence spectrophotometer (F-7000, HITACHI, Tokyo, Japan) at an excitation wavelength (Ex) of 335 nm and emission wavelength (Em) of 400 nm.

### 2.3. MG-Trapping Assay

In general, GKM3- and GKK2-fermented supernatants (1 mL) were reacted with 2 mM MG (1 mL) in a glass vial and were incubated at 37 °C for 1 h. After the reaction, 1 mL of 12 mM OPD was added to the sample vial, and the mixtures were incubated at 37 °C for 15 min to derivatize MG into 2-MQ. Finally, the mixtures were centrifuged (14,000× *g*, 5 min) and then subjected to high-performance liquid chromatography (HPLC) analysis to determine the amount of MG in each group [22]. The MG-trapping ability of the samples was determined based on the percent changes in the intensity of the MG peak of HPLC chromatograms in the sample groups compared to those in the control group.

### 2.4. Glycated Protein–RAGE Binding Assay

A CircuLex AGE-RAGE in vitro Binding Assay Kit (No. CY-8151, MBL Medical & Biological Laboratories Co. Ltd., Nagoya, Japan) was used to determine the inhibitory effects of GKM3- and GKK2-fermented supernatants on RAGE ligation, which is an important biological response in the onset and progression of various diseases [23]. The assay was carried out according to the manufacturer’s instructions. In brief, the GKM3- or GKK2-fermented supernatants was added to the diluted recombinant His-tagged sRAGE in the microplate pre-coated with AGE2-BSA for an hour. After the wells were washed, the horse radish peroxidase (HRP)-conjugated anti-His-tag antibody was added for another hour. The wells were washed out again, then the tetra methylbenzidine (TMB), a chromogenic subtract which could be catalyzed by an anti-His-tag antibody, was incubated for 15 min at room temperature. With the addition of a stop solution, the inhibitory effect on the AGE-RAGE interaction was evaluated by measuring the amount of His-tagged sRAGE2 on the wells under 450 nm absorbance.

### 2.5. Statistical Analysis

The data shown are the means of the results obtained from three independent experiments, and the statistical differences between the data of the sample groups and control group were evaluated by a Student’s *t* test using SPSS 18.0 software (IBM, Armonk, NY, USA). Significant differences were accepted at *p* < 0.05.

## 3. Results

### 3.1. Effects of GKM3- and GKK2-Fermented Supernatants on Early and Late Stages of Protein Glycation

**Figure 1:**
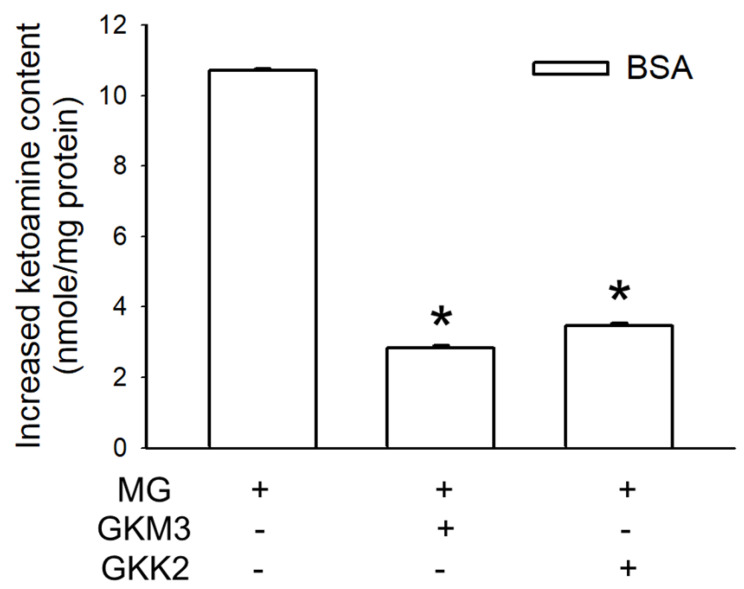
Effect of GKM3 and GKK2 on the formation of Amadori products in the early stage of protein glycation. Data unit was expressed as the increased nmole of ketoamines per mg of protein of samples. Data shown are means of results calculated by values of glycated BSA groups minus values of native BSA groups obtained from three independent experiments. * means statistical differences vs. data of the group of BSA treated with MG only. BSA: bovine serum albumin; MG: dicarbonyl-methylglyoxal.

One of the Amadori products, ketoamine, was detected and is shown in Figure 1 as the description of the early stages of protein glycation. The ketoamine content from the glycated BSA was 10.72 nmole/mg. With the addition of the GKM3-fermented supernatant, the concentration of ketoamine was decreased to 2.82 nmole/mg. A similar result was observed in the addition of the GKK2-fermented supernatant with a decrease in ketoamine content to 3.47 nmole/mg. These results suggested that both GKM3- and GKK2-fermented supernatants could inhibit the early stages of protein glycation by reducing the production of the Amadori product.

**Figure 2:**
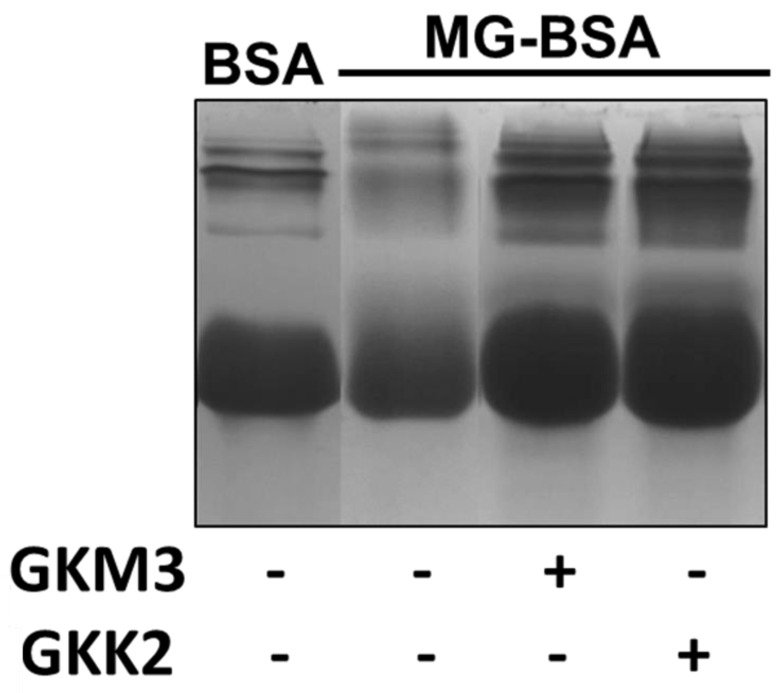
Effect of GKM3 and GKK2 on the SDS-PAGE results of products in the final stage of protein glycation. SDS-PAGE: sodium dodecyl sulfate polyacrylamide gel electrophoresis.

Furthermore, SDS-PAGE revealed the MG-trapping ability of the probiotic fermented supernatant (Figure 2). The protein cross-link aggregation occurred in the glycated BSA, which could be reduced by the presence of either GKM3- or GKK2-fermented supernatants. This suggests that the second stage of glycation reaction was also inhibited by the fermented supernatants from probiotics GKM3 or GKK2.

**Figure 3:**
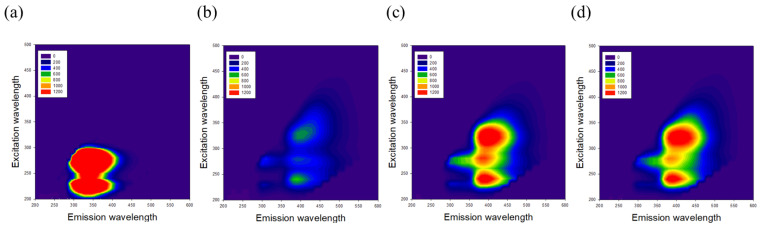
Effect of GKM3 and GKK2 on the 3-D fluorescent intensity of products in the final stage of protein glycation: (**a**) native BSA; (**b**) MG-BSA; (**c**) MG-BSA with GKM3; (**d**) MG-BSA with GKK2. Results shown are excitation–emission matrices (EEMs) of each group (excitation range: 200–500 nm; emission range: 200–600 nm).

The 3-D fluorescent intensity described the late stage of the protein glycation (Figure 3). There were significant differences between the fermented supernatants and the glycated BSA from the principle component analysis (PCA) (data not shown). This indicates that GKM3- and GKK2-fermented supernatants possessed abilities in inhibiting glycation.

**Figure 4:**
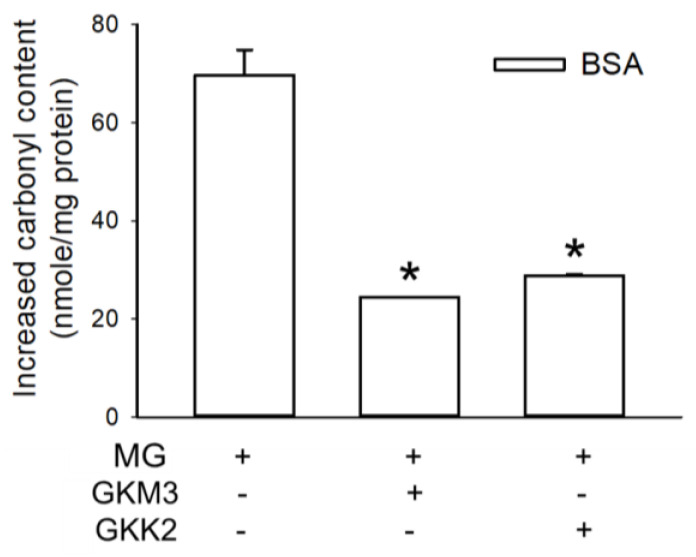
Effect of GKM3 and GKK2 on the content of carbonyl groups of products in the final stage of protein glycation. Data unit was expressed as the increased nmole of carbonyl groups per mg of protein of samples. Data shown are means of results calculated by values of glycated BSA groups minus values of native BSA groups obtained from three independent experiments. * means statistical differences vs. data of the group of BSA treated with MG only.

**Figure 5:**
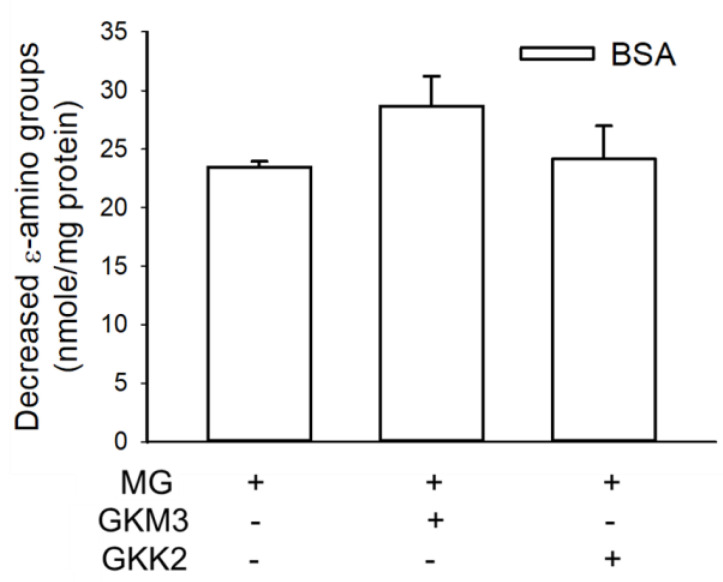
Effect of GKM3 and GKK2 on the content of ε-amino groups of products in the final stage of protein glycation. Data unit was expressed as the decreased nmole of ε-amino groups per mg of protein of samples. Data shown are means of results calculated by values of native BSA groups minus values of glycated BSA groups obtained from three independent experiments.

An analysis of changes in the specific functional groups on glycated BSA found that the carbonyl content increased 69.74 nmoles/mg (Figure 4) and the content of the ε-amino groups decreased 37.28% (Figure 5). The addition of the fermented supernatants from the probiotics GKM3 or GKK2 showed decreases in the carbonyl contents (*p* < 0.05). It is interesting that the addition of GKM3-fermented supernatants decreases the ε-amino groups but the addition of GKK2-fermented supernatants did not affect the decrease in the ε-amino groups a lot when compared to the glycated BSA. It is assumed that the supernatants from GKM3 and GKK2 possessed different function in changing the polypeptide structures. Furthermore, both GKM3- and GKK2-fermented supernatants could increase the free sulfhydryl content (data not shown) when compared to the glycated BSA.

### 3.2. Effects of GKM3- and GKK2-Fermented Supernatants on Formation of MG-Derived Advanced Glycation end Products and Related RAGE Ligation

**Figure 6:**
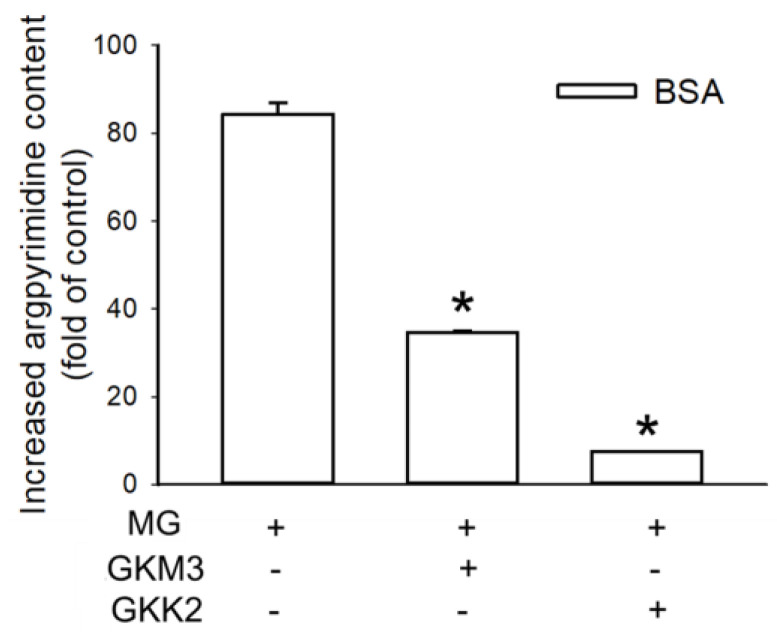
Effect of GKM3 and GKK2 on the formation of advanced glycation end products (AGEs) in the final stage of protein glycation. Data shown are means of ratio of values of glycated BSA groups versus values of native BSA groups obtained from three independent experiments. * means statistical differences vs. data of the group of BSA treated with MG only.

**Figure 7:**
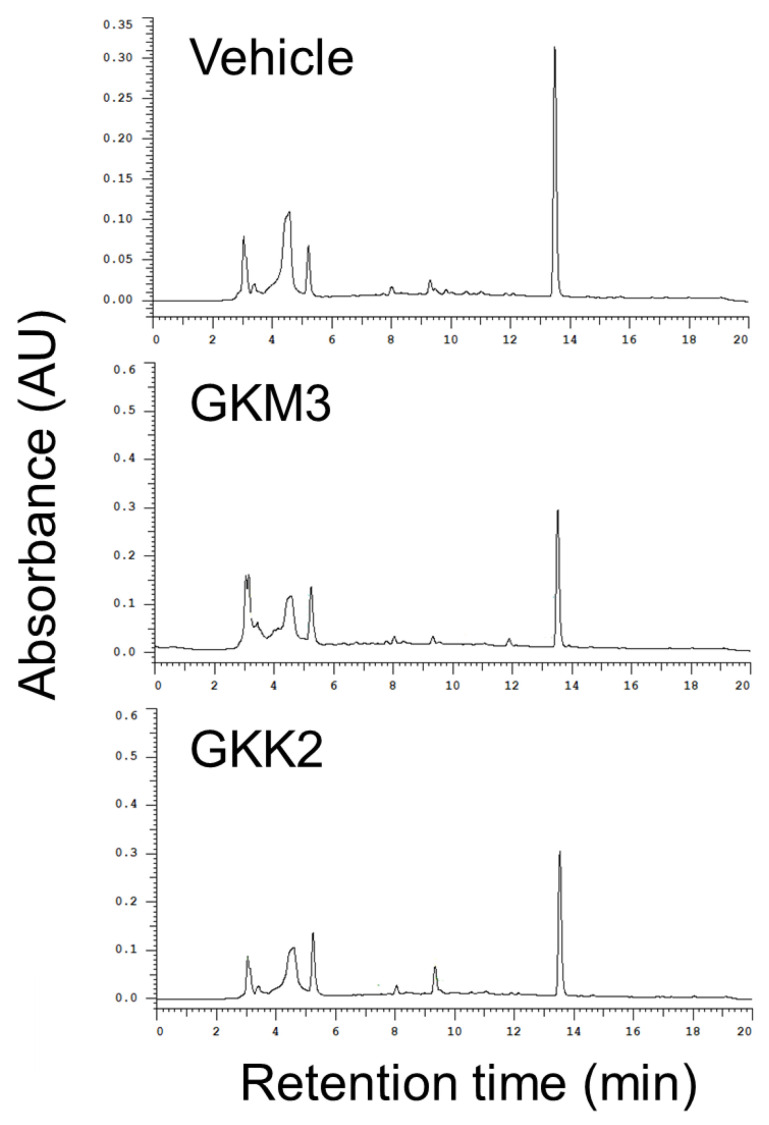
Methylglyoxal-trapping ability of GKM3 and GKK2. Data were expressed as the % of trapped methylglyoxal per 20 μL of samples. Data shown are means of results obtained from two independent experiments.

Argpyrimidine is one of the typical AGEs derived from MG. After the reaction among the BSA (5 mg/mL), glucose (10 mM), and MG (25 mM), the argpyrimidine was increased 84.32-fold. In the groups of GKM3- or GKK2-fermented supernatants, the contents of the argpyrimidine were significantly lower than the group of MG treatment only (*p* < 0.05) (Figure 6). Moreover, GKM3- or GKK2-fermented supernatants can decrease the MG intensity detected from HPLC analysis (Figure 7). This provided evidence that probiotic fermented supernatants suppressed the MG-derived AGEs.

**Figure 8:**
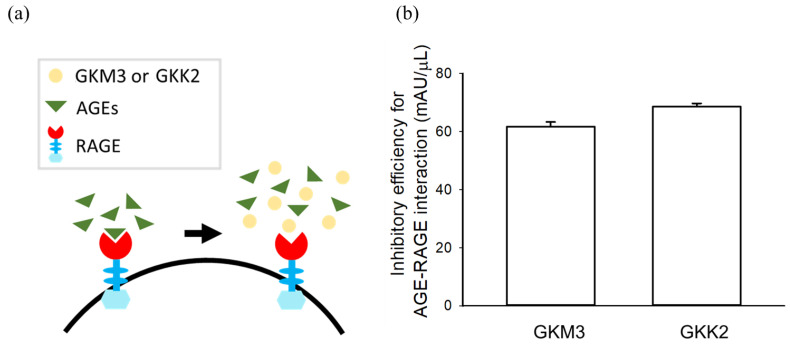
Effect of GKM3 and GKK2 on the ligation of RAGE with glycated proteins. (**a**) Scheme of inhibition of glycated proteins–RAGE ligation by GKM3 and GKK2 and (**b**) the experimental data. Data shown are means of results obtained from three independent experiments. RAGE: receptor for advanced glycation end products; AGE: advanced glycation end products.

Figure 8 shows the inhibitory efficiency of the AGE-RAGE interaction. Both probiotic fermented supernatants from GKM3 and GKK2 showed inhibitory rates for AGE-RAGE interaction of about 77% and 85%, respectively (Figure 8b). This suggests that probiotic fermented supernatants not only attenuated the glycation by inhibiting the production of Amadori products, MG derivatives, and glycated residues, but also by blocking the combination of the AGEs and RAGE to not trigger the downstream aging-related physiological cell pathways (Figure 8a).

## 4. Discussion

Ketoamine, also known as an Amadori product, is the main product in the early protein glycation stage [24]. The results indicated that GKM3- and GKK2-fermented supernatants significantly inhibited MG-induced ketoamine production in the early stage of the in vitro MG-BSA glycation model (*p* < 0.05) (Figure 1). MG can modify amino residues, specifically lysine and arginine, generating adducted amino residues, which can further result in protein cross-linking and the formation of macromolecules in the late stage of protein glycation [2]. In the present study, MG-induced protein cross-links can be found in the late stage of the in vitro MG-BSA glycation model as evidenced by the graphs of SDS-PAGE (Figure 2). According to the three-dimensional fluorescence spectrum, there are two major fluorophores in BSA (Figure 3a). One (Ex 275 nm/Em 340 nm) mainly results from the fluorescence of tryptophan and tyrosine residues, and another (Ex 225 nm/Em 340 nm) may be due to the π–π * transition of the polypeptide structures [17]. The BSA fluorescence was strongly modified after incubation with MG in the late stage of the in vitro MG-BSA glycation model (Figure 3b). In this regard, the MG-driven fluorescence quenching of tryptophan and tyrosine may result from an electron transfer or energy transfer reaction [25]. The MG-modified protein structure (Figure 2) and functional groups present in a protein including carbonyl groups and ε-amino groups (Figure 4 and Figure 5) can also affect the π–π * transition of the polypeptide structures. It was first demonstrated that GKM3- and GKK2-fermented supernatants can abrogate MG-induced protein cross-links (Figure 2) and changes in the carbonyl content of BSA (*p* < 0.05) (Figure 4), as well as MG-driven changes in the fluorescent characteristics of BSA (Figure 3c,d). This indicates that GKM3- and GKK2-fermented supernatants have great abilities on the prevention of protein glycation.

Similar results were also found in the previous study on *Lactococcus lactis* fermentates of cyanobacteria and microalgae [26]. Kaga et al. performed several bacteria-fermented edible algae with different anti-glycation activities, which was assumed from the different chemical composition of the algae species. In our pre-screened experiment of the lactic acid bacteria strains in the MG-BSA glycation model (data not shown), supernatants from different bacterial strains also performed different anti-glycation activities even though the fermentation process was conducted by using the same medium. That is, the bacteria itself possess different utilizations of the nutrition leading to diverse health benefits. As glycation includes the production of Amadori and an increase in the methylglyoxal concentration, some compounds from the food materials such as aloe (*Aloe sinkatana*), dill (*Anethum graveolens*), and pineapple guava (*Acca sellowiana*) have been reported to reduce the glycation process [27,28,29]. The GKM3- and GKK2-fermented supernatants could also play a role as inhibitors during the process. This provided the possibility of applying GKM3- and GKK2-fermented supernatants as health-promoting nutritional food materials.

Argpyrimidine is an MG-derived advanced glycation end product (AGE), which is implicated in aging-related cognitive dysfunction [30] and other disorders [2]. Gomes et al. found a high concentration of argpyrimidine in the patients with familial amyloidotic polyneuropathy, a neurologic disease [31]. This indicates that the potential suppression of the production of argpyrimidine might alleviate nerves’ relative symptoms. According to Figure 6, argpyrimidine residues significantly increased in MG-BSA (over 80 times to the native BSA) as evidenced by fluorescence analysis at Ex 335 nm/Em 400 nm; however, GKM3- and GKK2-fermented supernatants can significantly inhibit MG-driven increases in argpyrimidine. This may be related to the specific interaction of GKM3- and GKK2-fermented supernatants and the MG compound as evidenced by the changes in the intensity of the MG peak in the HPLC chromatogram of each group (Figure 7). Moreover, the inhibition of the content of argpyrimidine by the GKM3-fermented supernatant could explain the phenomenon of the memory retention that we observed in the age-accelerated mouse model in our previous publication [11]. In addition, the species *B. lactis* fermented supernatant reduced MG-induced argpyrimidine, which might lead to the improvement of the nerve conduction accompanied by a decreased expression of 8-OHdG or other oxidative markers in nerve cells, which is consistent with our observation in the SAMP8 mouse brain [10,32].

The receptor for AGEs (RAGE) is a multi-ligand receptor that has been demonstrated as the important role in the onset and progression of many diseases, such as diabetic complications, cardiovascular diseases, neuropathy, nephropathy, and cancer [23]. Therefore, AGE-RAGE ligation is the critical event in the progression of aging-related disorders [33]. Our study first demonstrated that GKM3- and GKK2-fermented supernatants could significantly inhibit AGE-RAGE interaction (Figure 8). This may be one of the mechanisms underlying the biological actions of the GKM3- and GKK2-fermented supernatants described in our previous studies [10,11]. The ligand–RAGE interaction activates NF-kappaB (NK-ĸB) and increased the expression of cytokines, adhesion molecules, and oxidative stress [34]. Consistent with other studies in probiotic fermented supernatants, liquid from fermented *Lactobacillus rhamnosus* showed an inhibitory effect on the expression of NK-ĸB in renal epithelial cells as well as other oxidative markers such as ERK, p53, and Caspase-3 [35]. We therefore assumed the fermented GKM3- and GKK2-supernatants presented the anti-aging effect by suppressing the relative oxidative markers involved with AGE-RAGE interaction.

## 5. Conclusions

Protein glycation is an important event during aging, which may increase AGE-RAGE interaction, leading to the occurrence of various diseases. In this study, GKM3- and GKK2-fermented supernatants were first found to significantly inhibit RCS-induced protein glycation, decrease the levels of argpyrimidine (the biomarker of aging diseases), and interrupt AGE-RAGE interaction, suggesting that lactic acid bacteria fermentates have great potential for the prevention of aging-related diseases.

## Data Availability

The data presented in this study are available on request from the corresponding authors.
